# Резолюция по итогам междисциплинарного экспертного совета «Когнитивное здоровье коморбидного пациента»

**DOI:** 10.14341/probl13398

**Published:** 2024-01-09

**Authors:** Е. В. Екушева, Л. А. Суплотова, Ф. Х. Дзгоева, О. В. Ремизов, З. Р. Гусова, М. Р. Бекуразова, А. П. Шепелькевич, Е. В. Ершова, К. А. Комшилова, А. Г. Кисиев, А. В. Анохина, В. В. Демидова, Н. В. Силина, Д. С. Рафикова, Д. М. Гасиева

**Affiliations:** Академия постдипломного образования Федерального государственного бюджетного учреждения «Федеральный научно-клинический центр специализированных видов медицинской помощи и медицинских технологий Федерального медико-биологического агентства»; Тюменский государственный медицинский университет; Национальный медицинский исследовательский центр эндокринологии; Северо-Осетинская государственная медицинская академия; Ростовский государственный медицинский университет; Северо-Осетинская государственная медицинская академия; Белорусский государственный медицинский университет; Национальный медицинский исследовательский центр эндокринологии; Национальный медицинский исследовательский центр эндокринологии; Северо-Осетинская государственная медицинская академия; Национальный медицинский исследовательский центр эндокринологии; Национальный медицинский исследовательский центр эндокринологии; Национальный медицинский исследовательский центр эндокринологии; Национальный медицинский исследовательский центр эндокринологии; Национальный медицинский исследовательский центр эндокринологии

**Keywords:** ожирение, коморбидность, профилактика, лечение, когнитивные функции, ВОЗ

## Abstract

29 сентября 2023 г. во Владикавказе состоялось заседание междисциплинарного экспертного совета «Когнитивное здоровье коморбидного пациента». Для снижения социального и экономического бремени когнитивных нарушений, все чаще выявляемых у коморбидных пациентов в Российской Федерации, необходимо внедрять социально значимые инициативы по своевременной диагностике и профилактике этих заболеваний, а также актуализировать современные подходы к лечению с учетом их многофакторного патогенеза и риска развития осложнений. По результатам проведенных в ходе экспертного совета научных докладов и дискуссии эксперты приняли решения по дальнейшему плану в рамках социально значимых инициатив по профилактике ожирения.

## Abstract (en)

On September 29, 2023, a meeting of the interdisciplinary expert council “Cognitive health of a comorbid patient” was held in Vladikavkaz. To reduce the social and economic burden of cognitive impairment, which is increasingly being detected in comorbid patients in the Russian Federation, it is necessary to introduce socially significant initiatives for the timely diagnosis and prevention of these diseases, as well as update modern approaches to treatment, taking into account their multifactorial pathogenesis and the risk of complications. Based on the results of scientific reports and discussions held during the expert council, experts made decisions on a further plan within the framework of socially significant initiatives for the prevention of obesity.

Когнитивные функции являются одними из наиболее сложных функций головного мозга, с помощью которых осуществляется взаимосвязь человека с окружающим миром, что позволяет реализовать такие процессы, как восприятие, мышление, внимание, речь, память и целенаправленную деятельность. Когнитивные нарушения (КН) часто встречаются в практике врачей разных специальностей, что обусловливает необходимость их своевременной диагностики и патогенетически обоснованной терапии. На сегодняшний день в мире почти у 50 млн людей отмечается деменция, и каждый год регистрируется около 10 млн новых случаев этого заболевания. По прогнозам специалистов, к 2030 г. количество людей, страдающих деменцией, удвоится, а к 2050 г. — утроится и будет составлять более 130 млн человек. Риск КН значительно повышается при наличии таких заболеваний, как артериальная гипертензия, сахарный диабет, ожирение, неалкогольная жировая болезнь печени и др., особенно при их сочетании, что часто наблюдается у коморбидных пациентов в клинической практике.

Ожирение является часто наблюдаемой и актуальной медико-социальной проблемой во всем мире и тесно связано с рядом сопутствующих заболеваний и патологических состояний. По данным Всемирной организации здравоохранения (ВОЗ), в 2016 г. более 1,9 миллиарда взрослых людей старше 18 лет имели избыточную массу тела, из которых 650 миллионов страдали ожирением. По данным исследования STEPS (англ. The WHO STEPwise approach to Surveillance — поэтапный мониторинг факторов риска ХНИЗ, разработанный ВОЗ), проведенного врачами-эндокринологами в Республике Беларусь в 2016–2017 гг., около 60% населения имеют избыточную массу тела, 25,4% пациентов — ожирение, в том числе висцеральное. При этом важно учитывать, что ожирение является коморбидным фактором для развития цереброваскулярной патологии и поражения нейрональных систем головного мозга, характеризующихся в первую очередь КН разной степени выраженности вплоть до деменции. У 95% пациентов с ожирением и избыточной массой тела отмечается снижение концентрации внимания, скорости обработки информации, объема оперативной и кратковременной памяти, нарушение процессов быстрого переключения и устойчивости внимания, а также исполнительного контроля. Причем эти изменения возникают уже с раннего возраста.

Для снижения социального и экономического бремени КН, все чаще выявляемых у коморбидных пациентов в Российской Федерации, необходимо внедрять социально значимые инициативы по своевременной диагностике и профилактике этих заболеваний, а также актуализировать современные подходы к лечению с учетом их многофакторного патогенеза и риска развития осложнений. Стоит отметить, что широкое использование таких малоинвазивных диагностических методов, как магнитно-резонансная томография, может способствовать усовершенствованию имеющихся диагностических алгоритмов ведения пациентов с КН различного генеза. Модификация образа жизни пациента является основой профилактики нарушения когнитивных функций и способствует улучшению качества жизни коморбидных пациентов.

По результатам проведенных в ходе Совещания научных докладов и дискуссии, было признано, что:

По результатам проведенных в ходе Совещания научных докладов и дискуссий было принято решение:

Резолюция одобрена и принята единогласно по результатам открытого голосования участников экспертного совета 29.09.2023 г.

## ДОПОЛНИТЕЛЬНАЯ ИНФОРМАЦИЯ

Источники финансирования. Работа выполнена по инициативе авторов без привлечения финансирования.

Конфликт интересов. Авторы декларируют отсутствие явных и потенциальных конфликтов интересов, связанных с содержанием настоящей статьи.

Участие авторов. Все авторы одобрили финальную версию статьи перед публикацией, выразили согласие нести ответственность за все аспекты работы, подразумевающую надлежащее изучение и решение вопросов, связанных с точностью или добросовестностью любой части работы.

Благодарности. Выражается благодарность Ванатовой Ольге Владимировне, Исаевой Умулкулсум Солтановне, Темирдашевой Наталье Михайловне, Барахоевой Розе Махмутовне, Славицкой Елене Семеновне, Камалову Камалу Гаджиевичу, Шихалиевой Инне Николаевне, Бабковой Наталье Валерьевне.

